# X-ray-Based Spectroscopic Techniques for Characterization of Polymer Nanocomposite Materials at a Molecular Level

**DOI:** 10.3390/polym12051053

**Published:** 2020-05-04

**Authors:** Dongwan Son, Sangho Cho, Jieun Nam, Hoik Lee, Myungwoong Kim

**Affiliations:** 1 Department of Chemistry and Chemical Engineering, Inha University, Incheon 22212, Korea; sonob99707@gmail.com (D.S.); skawl0430@gmail.com (J.N.); 2 Materials Architecturing Research Center, Korea Institute of Science and Technology, Seoul 02792, Korea; scho@kist.re.kr; 3 Division of Nano & Information Technology, KIST School, Korea University of Science and Technology, Seoul 02792, Korea; 4 Research Institute of Industrial Technology Convergence, Korea Institute of Industrial Technology, Ansan 15588, Korea

**Keywords:** polymer nanocomposite, qualitative analysis, quantitative analysis, X-ray photoelectron spectroscopy, energy-dispersive X-ray spectroscopy, X-ray absorption spectroscopy

## Abstract

This review provides detailed fundamental principles of X-ray-based characterization methods, i.e., X-ray photoelectron spectroscopy, energy-dispersive X-ray spectroscopy, and near-edge X-ray absorption fine structure, and the development of different techniques based on the principles to gain deeper understandings of chemical structures in polymeric materials. Qualitative and quantitative analyses enable obtaining chemical compositions including the relative and absolute concentrations of specific elements and chemical bonds near the surface of or deep inside the material of interest. More importantly, these techniques help us to access the interface of a polymer and a solid material at a molecular level in a polymer nanocomposite. The collective interpretation of all this information leads us to a better understanding of why specific material properties can be modulated in composite geometry. Finally, we will highlight the impacts of the use of these spectroscopic methods in recent advances in polymer nanocomposite materials for various nano- and bio-applications.

## 1. Introduction

Polymer nanocomposites comprise a soft polymeric matrix material and a dispersed filler material with the dimension in the nanometer scale, providing a heterogeneous system at a molecular level [1,2,3,4,5,6]. The type of the nanofiller is not limited; for example, inorganic or organic particles with different shapes including a sphere, cylinder, fiber, platelet, and so on [7,8,9,10,11,12,13,14]. In materials science and engineering, the nanocomposite materials are readily found due to their easiness to tailor the chemical and physical properties of different polymeric materials towards desired properties for target applications. One of the common examples is tuning thermal properties such as glass transition and degradation behaviors of polymeric materials by incorporating a range of fillers from hard oxide or metal nanoparticles to carbon materials such as carbon nanotubes and graphene [15,16,17,18]. The use of nanocomposite is not limited to only the improvement of physical properties: unique specific properties are intended to impart for resolving the challenges in certain applications. The formation of fibrous nanocomposites is an interesting and significant example: the combination of different polymers and functional fillers in a nanofibrous form enables organic, inorganic, and their hybrid functional nanofibers that can be applied in a number of applications from biological engineering such as drug delivery [19,20,21,22] and wound healing systems to energy engineering including photovoltaics and batteries [23,24,25,26].

To achieve these applications, three aspects should be considered, (i) design of the composite materials at a molecular level, (ii) synthesis and processing to realize the composites, and more importantly, (iii) acquiring important chemical and physical understandings for the components themselves as well as for the interfaces between filler and matrix which govern final target properties. For common carbon-based polymer nanocomposites, the easiest information to find is the atomic percentage of the elements in the nanocomposite, which provides information on the formation of the elements that make up the polymer nanocomposite. Beyond the chemical composition, the molecular structure has to be probed to comprehensively understand the system [10,27]. Furthermore, the physical state at the interface between the filler and polymer matrix has intrigued composite scientists and engineers [28,29,30,31,32], which ultimately leads us to complete structure–property relationships in composite materials for rational design towards desired applications. In these aspects, X-ray-based spectroscopic techniques have been highly relevant for a number of reasons including (i) accessibility to the core electron in a given material which is capable of qualitative and quantitative evaluation for almost all elements, their orbitals, and chemical bonds in common material platforms [33,34,35,36], (ii) feasibility of nondestructive analysis [37,38,39,40,41,42], (iii) accessibility to commercialized X-ray-based equipment, (iv) unnecessity of dissolution in organic solvents, and so on. In this review, we will focus on the fundamental principles and developed approaches and methods for qualitative and quantitative characterizations of polymer nanocomposites using widely utilized X-ray based spectroscopic techniques: X-ray photoelectron spectroscopy, energy-dispersive X-ray spectroscopy, and near-edge X-ray absorption fine structure. Finally, this review will highlight how these spectroscopic methods have made impacts on the advances in the understanding of created nanocomposite systems at a molecular level, i.e., the interface between an organic material and an inorganic material, towards attaining a variety of nano- and bio-applications. 

## 2. X-ray Photoelectron Spectroscopy

X-ray photoelectron spectroscopy (XPS) is one of the most well-known and widely used methods for the analysis of solid-state materials. It measures the kinetic energy of emitted electrons by photoelectric effects induced by X-ray illumination based on Einstein’s photoelectric effect [43]. The energy of released core electrons is one of the unique characteristics of the elements to obtain information on the number of elements as well as the chemical bonds in the system [44,45,46]. For this reason, XPS is also called electron spectroscopy for chemical analysis (ESCA). Since the photoelectron generated inside a solid sample can be scattered by the interaction with other electrons in the system, the mean free path of the photoelectron is very short, at several nanometers [47,48]. Thus, it is generally exploited for qualitative and quantitative analysis of the chemical structure and composition of the surface of the sample. It has the advantage that non-destructive analysis is possible due to its spectroscopic principle without damaging the samples. Therefore, the use of XPS is not limited as long as the interested sample is solid; examples include catalysts, semiconductors, and surface-modified areas such as thin films and coating [49].

In this section, the basic principle of photoelectron spectroscopy and analysis methods of polymer nanocomposite using photoelectron spectroscopy will be described. A quantitative analysis method for chemical composition, i.e., relative and absolute concentrations of certain elements and chemical functional groups near the interface between organic and inorganic materials will be discussed. A variety of additional techniques will be discussed to analyze other aspects of the materials, e.g., the depth of the surface and the perpendicular distribution of specific elements. We aim to also discuss detailed studies on an organic/polymeric thin film on a solid surface even if it is deviated from an exact definition of a nanocomposite, as those studies fundamentally elucidate a chemical state near the surface of nanofillers in a polymer matrix.

### 2.1. Principle of XPS

**Figure 1:**
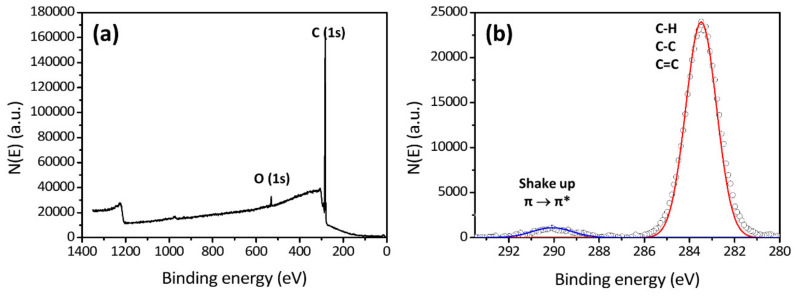
X-ray photoelectron spectroscopy (XPS) (**a**) survey spectrum and (**b**) C(1s) multiplex spectrum of polystyrene synthesized by atom transfer radical polymerization.

XPS is a method of analyzing the photoelectrons emitted by the photoelectric effect by irradiating an X-ray to a solid material. When the X-ray is irradiated to a solid sample, the energy of the photoelectron released from the specimen can be measured and the binding energy of the electrons at the core of a specific element in the material can be calculated. Since the binding energy of the core electrons is the inherent property in each element, the elemental composition of the sample can be analyzed. In general, Mg Kα (*hν* = 1253.6 eV) or Al Kα (*hν* = 1486.6 eV) X-rays are generally used to emit photoelectrons. As shown in Figure 1a, the XPS spectral shape of a sample is determined by the kind and quantity of core electrons composing the sample. The irradiated X-ray has high energy, enough to emit the electrons of the K shell whose energy level determines the energy of the photoelectrons (Figure 1a). At this time, the kinetic energy *E_k_* of the electron is defined by Equation (1):

*E_k_ = hν–E_B_–φ* (1)

where *hν* is the energy of the irradiated X-ray photon, *E_B_* is the binding energy of the photoelectron, and *φ* is the intrinsic work function of the spectrometer. From Equation (1), the binding energy can be calculated with the known X-ray photon and the measured *E_k_*. When energy is applied to the element, the excited electron tends to depart from the nucleus and reaches the point at which the binding energy between the nucleus and the electron becomes zero, and escapes the restrained status from the nucleus to outside of the element. The energy level is defined as the Fermi level. Thus, the binding energy of the photoelectron is the energy difference between the Fermi level and the orbitals of the originated electron. The binding energy is a unique characteristic of each element, as it is determined by the attraction between the nucleus and the electron, relying on the number of protons in the nucleus. Light sources with high energy are required to emit core electrons of the K shell having a very large binding energy by the photoelectric effect. Therefore, Al Kα or Mg Kα are generally used as the X-ray light source. The further away the electron is from the atomic nucleus, the weaker the attraction between the two, and thus the binding energy decreases. On the contrary, as the distance between the electron and the atomic nucleus gets closer, the binding energy increases. Therefore, it is possible to distinguish a specific orbital of a specific element present in a sample by measuring binding energy. Since the binding energy of the specific orbital of each element varies slightly according to various chemical bonding states, it is also possible to analyze the binding state of specific atomic orbitals of specific elements.

Based on this principle, we can obtain the spectrum of the intensity of the photoelectrons generated as a function of the binding energy by the characterization of the polymer nanocomposite using XPS. Figure 1 shows the XPS spectrum of a polystyrene thin film. A spectrum with a wide range of binding energy, 0 to ca. 1400 eV, is called “a survey spectrum”, and a high-resolution spectrum with a specific range of binding energy for the desired element is called “a multiplex spectrum”. The survey spectrum shows which elements exist in a given sample and their relative proportions, i.e., the atomic percent. Further, the shape of a multiplex spectrum of the element of interest provides its detailed chemical bonding state. 

While the photoelectrons emitted by X-ray irradiation move to escape the surface from the inside of the sample, they are subjected to continuous inelastic collisions with the electrons of the elements inside the sample. Photoelectrons in inelastic collisions lose their energy as well as the information about the binding energy of the core electrons. The physical quantity that describes this phenomenon is called the inelastic mean free path (*λ*), which is defined as the average path that the electrons travel to make inelastic collisions with other electrons in the sample. The typical *λ* of a photoelectron emitted by an X-ray is several nanometers, depending on the type of the sample and the kinetic energy of the photoelectron. Theories dealing with the peak intensity of the XPS spectrum describe that more than 95% of electrons lose their energy by experiencing inelastic collisions within a distance of 3*λ*. Therefore, only the information from the depth within 3λ, approximately 10 nm in the case of the polymeric materials, can be obtained in a typical XPS analysis; thus this depth may be referred to as the signal detection depth or the sampling depth. It should be noted that it is not possible to analyze the composition inside the sample deeper than the sampling depth. To overcome this, vertical distribution analysis using etching can be performed, which will be described in detail later.

### 2.2. Identification of Elements and Chemical Bonds

**Figure 2:**
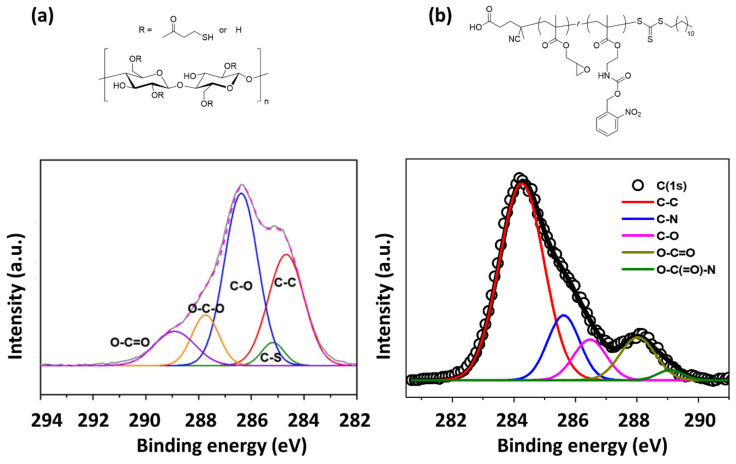
Deconvoluted C(1s) multiplex spectra of (**a**) a thiol-functionalized cellulose nanofiber mat (Reproduced from Reference [50] with permission from Elsevier) and (**b**) a photo-crosslinked thin film of poly(2-((((2-Nitrobenzyl)oxy)carbonyl)amino)ethyl methacrylate-*r*-glycidyl methacrylate) (Reproduced with permission from Reference [51]; Copyright (2015) American Chemical Society).

The first step in the XPS analysis of polymer nanocomposites is usually to obtain a survey spectrum to determine what elements are present. In general, the peak positions of elements of typical interest identified in organic polymer nanocomposites are approximately C(1s) at 285 eV, N(1s) at 398 eV, O(1s) at 531 eV, F(1s) at 685 eV, Si(2p) at 99 eV, S(2p) at 164 eV, S(2s) at 228 eV, P(2p) at 130 eV, P(2s) at 188 eV, Cl(2p) at 200 eV, Cl(2s) at 271 eV, and Br(3d) at 69 eV. After identifying the elements in the survey spectrum, the acquisition of a high-resolution spectrum of a certain element is conducted to obtain a multiplex spectrum and analyze its chemical environment. Since the binding energy of the core electron is a unique value of the element, XPS is a good method for qualitative analysis. The binding energies of elements vary slightly depending on their surrounding chemical environment, that is, their atomic bonding state or lattice site. In the case of bonding in organic materials, an atom with a larger electronegativity within a small or large molecule reduces the electron density of an atom with a smaller electronegativity. The electronic repulsive force in an atom with smaller electronegativity decreases while the total charge of the nucleus is kept as constant. Therefore, the atomic nucleus more strongly attracts electrons, which results in an increase in the binding energy, the so-called chemical shift. The chemical shift widens the multiplex spectrum which allows us to determine the type of chemical bonds by the deconvolution of the peak of interest. As the type of chemical bond of carbon element in polymer nanocomposites is highly important to probe their chemical composition, the high-resolution spectrum of C(1s) is carefully examined. Table 1 summarizes the position and assignment of characteristic peaks in C(1s) spectrum. It is noted that the position is often affected by the conditions of samples and measurements; for example, the accumulation of positive charge upon the photoemission of electrons on an electrically insulated surface, i.e., charging effect, the peak positions are often deviated from the representative values and therefore, calibration by positioning C-C (or C-H) peak in C(1s) region at 285.0 eV is necessary for the peak interpretation. Figure 2 shows a clear emergence of peaks in correspondence to the functionality in a given chemical structure in C(1s) multiplex spectra with an appropriate deconvolution, highlighting the ability to resolve complex chemical structures [50,51]. These analyses have been routinely performed in a number of studies on polymer nanocomposite materials. Here, we introduce the use of the XPS analysis in recent notable polymer nanocomposite studies for various target applications.

**Figure 3:**
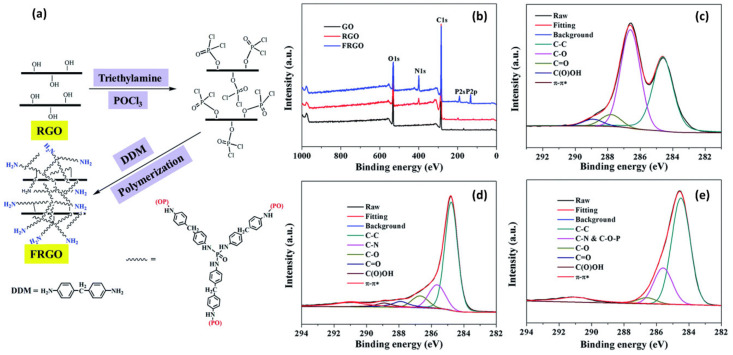
(**a**) Preparation of functionalized reduced graphene oxide (FRGO) nanocomposites. (**b**) XPS survey spectra of graphene oxide (GO), reduced graphene oxide (RGO) and FRGO; high resolution C(1s) XPS spectra of (**c**) GO, (**d**) RGO and (**e**) FRGO (Reproduced from Reference [36] with permission from the Royal Society of Chemistry).

Hu et al. utilized an XPS analysis to monitor each step for the preparation of functionalized reduced graphene oxide (FRGO) [36]. The FRGO was prepared by chemical reduction of graphene oxide followed by polymerization of 4,4′-diaminodiphenylmethane on POCl_2_-treated reduced graphene oxide (RGO) (Figure 3a). The survey spectra revealed the newly appeared N(1s) for RGO and P(2s) and P(2p) for FRGO, which were attributed to the nitrogen doping of graphene during the reduction reaction of graphene oxide (GO) and the wrapped flame retardants on the RGO surface, respectively (Figure 3b,c). In the deconvoluted C(1s) multiplex spectrum of RGO in Figure 3d, a decrease in the relative peak areas of oxygen-containing groups is observed, indicating the successful removal of oxygen-functional groups during the reduction reaction. The diminish of C=O and O-C=O bands in FRGO could be attributed to the wrapping effect of flame retardants in the high-resolution C 1s spectrum of FRGO (Figure 3e).

**Figure 4:**
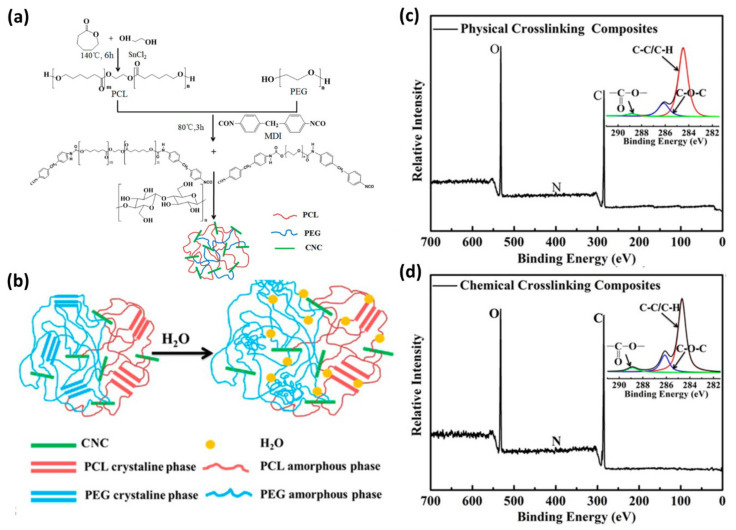
Shape memory process of PEG-PCL-CNC thermo-responsive nanocomposite. (**a**) The scheme showing the synthetic route for the PEG-PCL-CNC nanocomposite. (**b**) Shape memory mechanism of the PEG-PCL-CNC nanocomposite water responsiveness. XPS survey spectra of the (**c**) physically cross-linked nanocomposite and (**d**) chemically cross-linked nanocomposite PEG(60)-PCL(40)-CNC(10) (inset images show C(1s) multiplex spectra) (Reproduced with permission from Reference [33]; Copyright (2015) American Chemical Society).

Figure 4 shows the preparation and its characterization of cellulose nanocrystal (CNC)-crosslinked shape-memory polymer nanocomposites [33]. The polymer nanocomposites were prepared by condensation of separately prepared polycaprolactone (PCL)- and poly(ethylene glycol)-based prepolymers with CNCs (Figure 4a). Depending on the participation of hydroxy groups on the surface of the CNCs in the condensation reaction, they were crosslinked with the polyurethane chains either physically or chemically. The main chain elements and the carbon-based bonds of the nanocomposites were characterized by XPS analysis. As expected, the polymer nanocomposites consisted of carbon, oxygen. The high-resolution C(1s) spectra indicated that the relative amount of O-C=O in the composites was higher in the chemically crosslinked sample than in the physically crosslinked one. The results support the successful condensation reaction between CNC and the prepolymers. 

**Figure 5:**
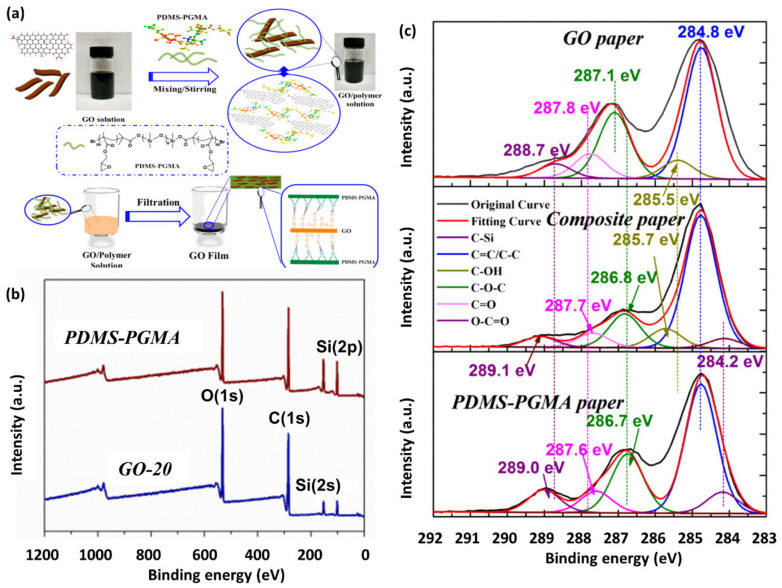
(**a**) Schematic illustration for the preparation of GO/polydimethylsiloxane-poly(glycidyl methacrylate (PDMS−PGMA) composite paper achieved through mixing GO solution with PDMS−PGMA triblock copolymer, (**b**) survey spectra of PDMS-PGMA and GO/PDMS-PGMA composite papers, and (**c**) deconvoluted C(1s) XPS spectra of GO, PDMS-PGMA, and GO/PDMS-PGMA composite films. (Reproduced with permission from Reference [34]; Copyright (2016) American Chemical Society).

Zhang et al. used XPS analysis to support the adhesive property of polydimethylsiloxane-poly(glycidyl methacrylate (PDMS-PGMA) in graphene oxide (GO)/polymer nanocomposite papers [34]. The nanocomposite papers were prepared by filtration of mixing solution of PDMS-PGMA and GO (Figure 5a). Both survey scans of PDMS-PGMA and GO/PDMS-PGMA composite papers exhibited four peaks corresponding to C(1s), O(1s), Si(2s), and Si(2p), which indicated the co-existence of the polymer and GO in the composite papers. The deconvoluted high-resolution C(1s) spectra (Figure 5c) showed a peak corresponding to C-Si only in PDMS-PGMA paper, but not in the GO paper, suggesting C-Si peak can be a standard peak to distinguish the polymers. Also, the peaks corresponding to C-OH, C-O-C, C=O, and O-C=O groups shifted among GO, PDMS-PGMA, and GO/PDMS-PGMA papers, providing evidence for hydrogen bonding in the composite papers.

**Figure 6:**
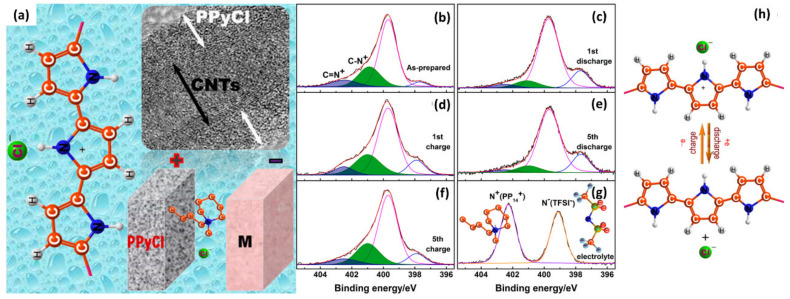
(**a**) Schematic illustration of PPyCl@CNTs cathode (a-g) XPS N(1s) multiplex scan of (**b**) as-prepared PPyCl@CNTs cathodes, and subsequent (**c**) first discharge and (**d**) first charge, (**e**) fifth discharge and (**f**) fifth charge, and (**g**) the electrolyte itself. (**h**) The charge/discharge mechanism in the portion of PPyCl. (Reproduced with permission from Reference [35]; Copyright (2017) American Chemical Society).

Shen et al. demonstrated charge/discharge behavior at polypyrrole chloride@carbon nanotubes (PPyCl@CNTs) cathode in the chloride ion battery by using XPS analysis [35] (Figure 6). The PPyCl@CNTs nanocomposite materials were prepared by FeCl_3_-catalyzed oxidation polymerization of pyrrole in the aqueous dispersion of CNTs. The high-resolution N(1s) spectra of the composite cathode materials were composed of four peaks at 397.7, 399.7, 401, and 402.5 eV, corresponding to C-N, -NH-, (polaron, C-N^+^), and (bipolaron, C=N^+^), respectively (Figure 6b–g). Upon discharge, the intensities of C-N^+^ and C=N^+^ peaks decreased, indicating the reduction of the amount of N^+^ species and the de-doping or deintercalation of Cl in the composite cathode. The N^+^ species were recovered in the following charge step. The reversible electrochemical reaction was demonstrated by the repeated increase and decrease in N^+^ species peaks during five cycles. This study suggested an analytical method for the illustration of electrochemical reactions in battery applications. 

### 2.3. Quantitative Analysis

#### 2.3.1. Elemental or Atomic Percentage

In the survey spectrum or the multiplex spectrum, the areas of the peaks of all the elements composing the polymer nanocomposite can be obtained by integrating the peaks in the spectrum. The atomic percentage can be calculated using the following equation. For the polymer nanocomposite comprising C, N, O, and S, the atomic percentage of N is analyzed with the Equation (2):

(2) $$\mathrm{Atomic}\%(N)=\frac{\frac{A\left(N\right)}{s\left(N\right)}}{\frac{A\left(C\right)}{s\left(C\right)}+\frac{A\left(O\right)}{s\left(O\right)}+\frac{A\left(S\right)}{s\left(S\right)}+\frac{A\left(N\right)}{s\left(N\right)}}$$

*A(X)* and s(*X*) are the integrated area of the XPS peak the sensitivity factor for the element *X*. In the XPS analysis, the amount of photoelectron generated varies from element to element; therefore, the sensitivity factor is used to compensate for the difference. Theoretically, when *s(F(1s))* is 1, *s(C(1s))* has a sensitivity factor of approximately 0.25. The factors are used to compensate for the integrated area to obtain absolute intensity, and then the quantitative analysis is feasible, although it is noted that the sensitivity factor differs from each equipment. 

#### 2.3.2. Density (or Concentration) Analysis of Specific Elements

The elemental percentage described above is the relative ratio of each element, not the absolute number of specific elements. Sometimes, measuring the absolute number of specific elements through XPS analysis is important depending on the target system. For example, Sweat et al. analyzed the absolute density of the surface initiators using XPS when synthesizing the polymer brushes on an inorganic solid surface through surface-initiated polymerization using atom transfer radical polymerization (ATRP) and associated them with the chain density of the resulting brushes [52,53], suggesting that XPS is an important analysis technique to achieve the desired chain density.

The intensity of photoelectrons occurring at a specific point in the polymer nanocomposites after X-ray irradiation is expressed as *I_0_*, *d* is the depth of occurrence point, *θ* is the detection angle of the detector, and *λ* is the inelastic mean free path of the photoelectron, then *I_S_*, the intensity of the photoelectron escaping from the surface, is expressed as Equation (3).

(3) $$I_{s}=I_{0}e^{-d/\left(\lambda ,coscϴ\right)}$$

Since some of the photoelectrons are scattered by other electrons inside the organic layer, the intensity of the XPS signal decreases as *d* increases and *λ* decreases. Equation (3) is the basis for quantitative analysis by analyzing the XPS spectrum. When an organic including the initiator group on an oxide surface on top of silicon, and its thickness is sufficiently thin, the photoelectrons are emitted from those three layers by X-ray irradiation, and these electrons are released into three routes as follows:

1. Electrons released from silicon
2. Electrons emitted from SiO_2_
3. Electrons released from an element in an organic layer.

In the case of 3, the electrons from the organic layer were represented by those emitted from the Bromine in the ATRP initiator. The intensity of the XPS signal of the electrons released from Si in SiO_2_ expressed in Equation (4) using Equation (3):

(4) ISi,SiO2=NSi,SiO2·∫0Toxe−zλ Si,SiO2cosϴdz

where *N_Si,SiO2_* is the number density of Si in native SiO_2_, *Tox* is the thickness of native SiO_2_ and *λ_Si,SiO2_* is the inelastic mean free path of the photoelectrons emitted from Si in SiO_2_. The released electrons pass through the polymer thin film and then completely escape from the surface to enter the detector. As a result, Si(2p) peaks occur at 101.5–104.5 eV. Using Equation (3), the intensity of the finally detected signal is given as Equation (5) by calibrating the inelastic collision of the photoelectron as it passes through the organic layer:

(5) ASiSSi=NSi,SiO2·e−Lλ Si,organiccosϴ·∫0Toxe−zλ Si,SiO2cosϴdz

where *A_Si_* refers to the integral area of the peak corresponding to Si of SiO_2_ from Si(2p) peak, *S_Si_* is the sensitivity factor of Si(2p), *L* is the thickness of the polymer thin film, and *λ_Si,organic_* is the inelastic mean free path when electrons released from silicon pass through the organic layer. Electrons released from a specific element (X) of the organic layer pass through the organic thin-film layer and arrive at the detector. The integrated area of the particular peak is given in Equation (6):

(6) AxSx=Nx.organic·∫0Le−zλ x,organiccosϴdz

where *A_x_* is the integrated area of the X peaks, *S_x_* is the sensitivity factor of X, *N_x,organic_* is the number density of X in the polymer thin film, and *λ_x,organic_* is the inelastic mean free path when electrons emitted from X pass through the organic layer. The ratio of Equation (5) to (6) can be induced as follows:

(7) AsiSsiAxSx=NSi,SiO2·e−Lλ Si,organiccosϴ·∫0Toxe−zλ Si,SiO2cosϴdzNx,organic·∫0le−zλ x,organiccosϴdz

*N_x,organic_* is then given as follows:

(8) Nx,organic=AxSxAsiSsi·NSi,SiO2·e−Lλ Si,organiccosϴ·∫0Toxe−zλ Si,SiO2cosϴ∫0Le−zλ x,organiccosϴ

which can be calculated from the integrated area of X on the XPS spectrum, that of the parts corresponding to SiO_2_ in Si(2p) peak, sensitivity factor of each element, the thickness of the organic layer and the native SiO_2_, and the number density of Si in native SiO_2_. *λ* of electrons in organic matter or oxide compound according to the kinetic energy of the photoelectron can be easily found in the literature [54,55,56]. In Sweat et al., the number density of Br after thermal annealing to introduce the organic layer containing ATRP initiators was 1.86 ± 0.12 Br atoms/nm^3^. With this result, the surface chain density of the polymer grown from the surface was calculated as 0.80 ± 0.06 chains/nm^2^. This is an example suggesting the potential use of XPS analysis in the synthesis of an organic thin layer on a solid surface, in that XPS, quantitative analysis should be beneficial to determine the amount of relevant element near the interface between two components in nanocomposite materials.

### 2.4. Angle-Resolved XPS

**Figure 7:**
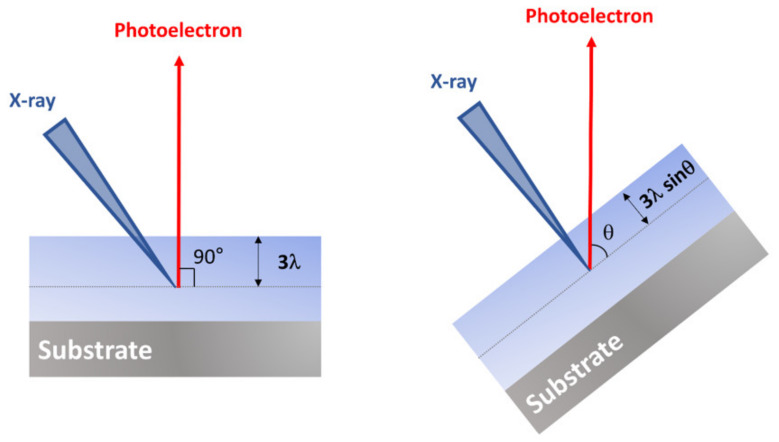
The changes in the effective photoelectron escape depth in angle-resolved XPS.

As mentioned earlier, the effective escape depth is correlated to the actual sampling depth, which is the function of the inelastic mean free path, the characteristic of the material itself. However, the various information of samples can be obtained by changing the incident angle. Tilting samples to have the angle to the surface as θ, the effective escape depth changes from 3*λ* to 3*λ*sinθ (Figure 7). As a consequence, the detectable depth dramatically reduces to a smaller extent, and an XPS signal from the area closer to the surface, that is, the more surface-sensitive chemical composition can be obtained. This method is called angle-resolved XPS (ARXPS) analysis. By using this method, the chemical structure of the surface can be easily understood. When the thickness of polymer thin film is less than 3*λ*, non-destructive vertical chemical composition and distribution of the material surface also can be analyzed. 

**Figure 8:**
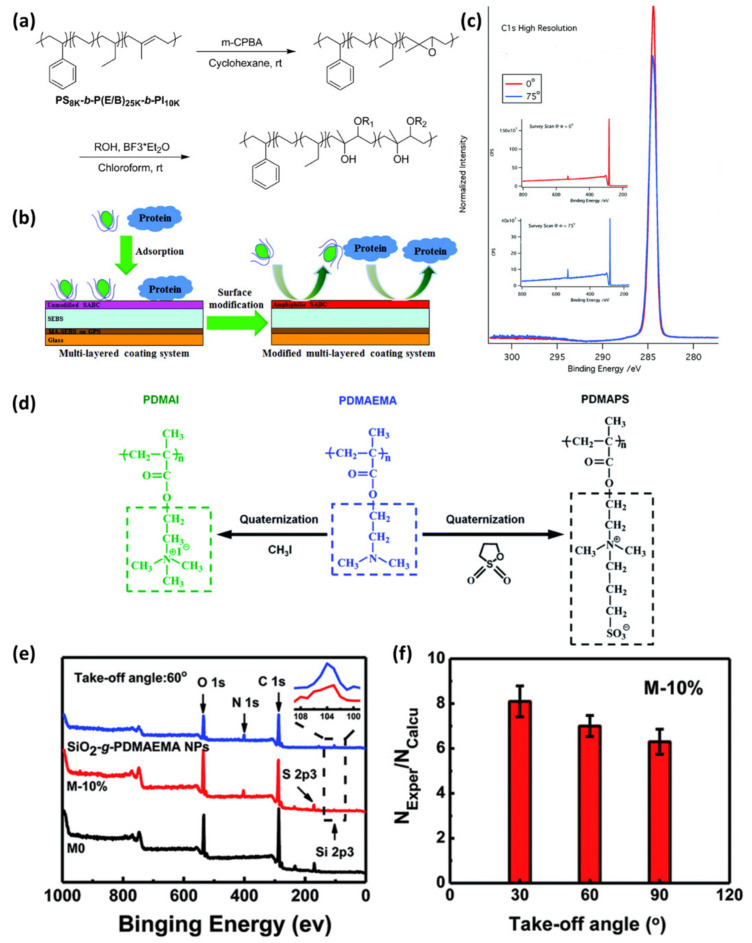
The use of angle-resolved XPS for surface active materials: (**a**–**c**) the surface-active block copolymers (SABCs) using triblock copolymer (Reproduced with permission from Reference [38]; Copyright (2012) American Chemical Society) and (**d–f**) the nanocomposite of polymer-grafted SiO_2_ nanoparticle in polyethersulfone matrix (Reproduced from Reference [40] with permission from the Royal Society of Chemistry).

This method is particularly beneficial for surface active materials. As a conventional example, Figure 8 shows the C(1s) signal of the surface-active block copolymers (SABCs) thin film, utilized for resistance to biofouling surface, at two tilting angles using ARXPS analysis technique [38]. Protein adsorption and resistance to biofouling polymers were synthesized using hydrophobic aliphatic (propylene oxide-derived groups or linear hydrocarbon) and hydrophilic poly(ethylene glycol) (PEG) side chains. The coatings were prepared by the polyisoprene (PI) block of PS-b-P(E/B)-b-PI triblock copolymer. Amphiphilic SABCs, a mixture of hydrophobic and hydrophilic side chains, was selectivity attached to hydrophobic aliphatic or PPG alcohols and hydrophilic PEG alcohols via Lewis acid-catalyzed ring-opening of the epoxidized isoprene block. Figure 8c shows the XPS multiplex C(1s) and survey spectra of the amphiphilic SABCs acquired with two different electron emission angles (0° and 75°) compared to the surface normal. The side chain of SABCs shows a peak at 285 eV of carbon bonded only to C and H, indicating that P(E/B) block of the SABCs has formed the surface as well. This type of study can also be found in a surface-active nanocomposite material. Zhu et al. reported the use of quaternary ammonium containing polymer brush grafted silica nanoparticle as a nanofiller in antifouling and anti-bacterial polyethersulfone membrane [40]. The ARXPS results in Figure 8e show the increase in N(1s) intensity when the angle is lowered, confirming that the density of the nanofiller exhibiting quaternary ammonium functional group near the surface is higher than inside of the membrane material and therefore, an improved surface activity correlated to antifouling and anti-bacterial performance is expected.

The examination of morphological behaviors of polymer thin film on a solid substrate is another example of ARXPS analysis. The interaction between block copolymer and the solid surface governs the phase segregation behavior and, hence, the effective modulation of morphology and orientation of the block copolymer. It can be controlled by (i) the solvent annealing process [57] and (ii) the surface reconstruction process of block copolymers. As a result, the fabrication of nanopores by selective swelling of one block in perpendicularly oriented structured block copolymers is possible [58]. In these studies, ARXPS studies effectively confirm the preference of one block to the surface rather than the interface between the copolymer and the solid surface, which led to the chemical composition changes along the z-direction.

### 2.5. Depth Profiling by Etching

A further examination inside a given sample deeper than the sampling depth of 3*λ* is not feasible in XPS analysis. To overcome this shortcoming and analyze the deeper portion of the sample, the depth profiling approach has been developed, a method continuously measuring the XPS spectrum while etching the specimen using an ion gun. Irradiating an ion beam which does not give chemical changes to samples, e.g., C_60_ cluster beam or argon cluster ion beam, can induce etching due to physical sputtering rather than chemical sputtering accompanying undesired chemical reactions. X-ray irradiation on the etched surface provides the information of chemical composition, qualitative and quantitative analysis within a deeper level of a sample. These acquired spectra can be plotted by adding a time axis, providing a three-dimensional spectrum, which is converted to the three-dimensional information of the sample by converting time to etched depth with an etch rate. Although this is a very powerful method to analyze the full depth range of a sample, it is inevitably destructively performed by damaging the surface, and the specimen can no longer expect an initialized structure at the end of the analysis.

**Figure 9:**
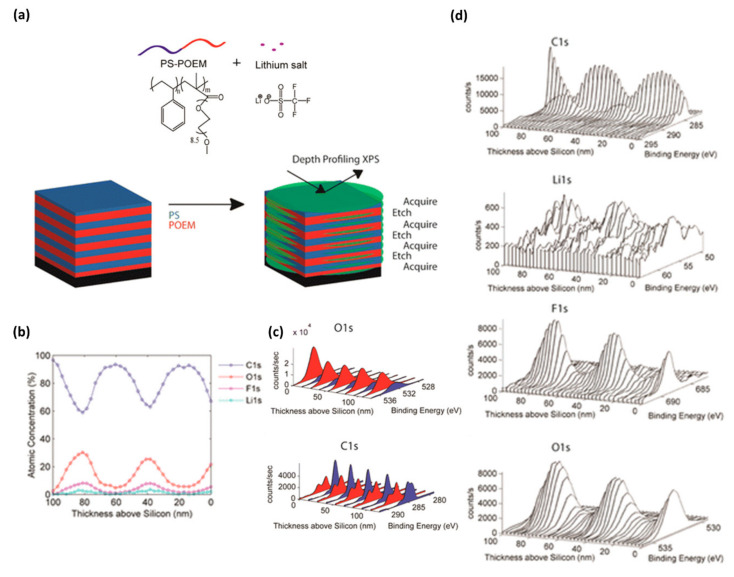
(**a**) Schematic illustration of depth profiling of a lamellae-structured polymer nanocomposite with block copolymers and Li-ions. (**b**) Atomic percentage vs thickness plots of F(1s), C(1s), O(1s), and Li(1s). (**c**) Photoelectron spectra of O(1s), and C(1s). (**d**) Thickness dependent XPS spectra of C(1s), Li(1s), F(1s), and O(1s). (Reproduced with permission from Reference [37]; Copyright (2015) American Chemical Society).

Figure 9 is an example of depth profiling analysis for the location of specific molecules within polymer composites. A polystyrene-*b*-poly(oligo(oxyethylene)methacrylate) (PS-*b*-POEMA) block-copolymer composite mixed with a small molecule containing Li-ions was coated on a silicon substrate to form a lamellae structure parallel to the substrate. XPS analysis with C_60_ cluster ion beam etching was used to determine where Li-ions exist within the vertical structure [37]. As shown in Figure 9, Li (1s) photoelectron was strong in the region of the POEMA block with the abundant O(1s) atoms. This study provides the potential of XPS analysis for the nanostructural analysis of polymer nanocomposites as well as the field of application of Li-ion/polymer nanocomposite-based batteries.

XPS analysis with the depth profiling method can also be used in medical engineering such as the proliferation and differentiation of stem cells. Schmitt et al. used XPS analysis to confirm the functionalization of peptides [39]: a thermally cross-linked polymeric thin film containing azlactone was functionalized with RGD (Alginin-Glycine-Aspartic acid) peptides with the terminal primary amine or thiol functional group, which was useful for culturing human mesenchymal stem cell. The location of functionalization, whether it was on surface or inside of the film, was analyzed by using XPS analysis with argon ion beam etching. They measured the atomic percentage as a function of etching time, and N(1s) photoelectrons were emitted in the whole area of the thin film. This observation provides the mechanistic insight on the functionalization reaction, suggesting the immobilization reaction occurred in the entire organic rather than only on the surface. This is a notable observation as it can be applied to the post-processing chemical modification of the polymer nanocomposite. In this aspect, XPS can be used to study the correlation between chemical reactions and swelling behaviors of polymeric material, i.e., the depth the reactant molecule reaches with the variation in the size of the reactant molecule. Barbey et al. employed depth profiling with XPS to determine how deep reactant molecules penetrate and encounter to make the successful reaction of primary amine group with epoxy group in poly(glycidyl methacrylate) (PGMA) [41]. Propyl amine was used as a small reactant molecule containing primary amine, and XPS analysis was conducted with etching the polymer thin film with C_60_ cluster ion beam sputtering. Since the PGMA does not have a nitrogen atom, the degree of reaction can be tracked through the monitoring of the N(1s) spectrum. This study gave detailed information on the penetration of propyl amines into the PGMA, which is an important study to find a correlation between the molecular size and the diffusion within the polymer matrix.

**Figure 10:**
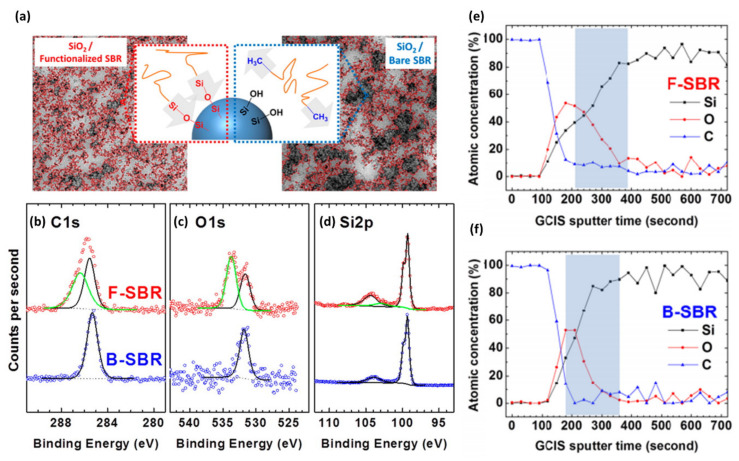
(**a**) TEM images, (**b**–**d**) XPS multiplex spectra, and (**e**–**f**) depth profiling results of the SiO_2_/functionalized styrene-butadiene (SBR) polymer (F-SBR, red) and SiO_2_/bare SBR (B-SBR, blue) nanocomposites. (Reproduced with permission from Reference [42]; Copyright (2015) American Chemical Society).

Figure 10 shows an example of XPS analysis on the surface chemistry at the interface of silica nanofillers and polymer matrix [42]. Hydrophilic nanofillers have been widely used to tune the mechanical properties of the polymer matrix. However, the difference in surface energy between hydrophilic nanofillers and hydrophobic polymer matrix results in inhomogeneous dispersion of the nanofillers within the matrix. To overcome this problem, Parekh et al. functionalized the hydrophilic silica nanoparticles with silanol end-functionalized styrene-butadiene rubber (SBR) polymers, expecting a reduction in the surface energy of the nanoparticles and homogeneous distribution of them within the SBR matrix. Comparing the dispersion of silica nanoparticles with and without surface functionalization, TEM images revealed that homogeneous distribution of the SBR-tethered silica nanoparticles within the matrix while unmodified silica nanoparticles aggregated in the matrix (Figure 10a). In this study, depth-profiled XPS analysis was used to distinguish the interaction between silica nanoparticles with SBR polymer chains either in chemisorption or in physisorption. As a model system, the atomic percentage of the elements at different depths of silanol end-functionalized and bare SBR polymer films on Si wafers were obtained during repeated argon cluster sputtering to define the interface (Figure 10e–f). At the specific etching depth representing the interface between SBR polymers and the substrate, high-resolution spectra of C(1s), O(1s), and Si(2p) were compared (Figure 10b–d). The thin film of silanol end-functionalized SBR polymer exhibited additional C-Si-O, Si-O-R/Si-O-Si, and C-Si-O-Si at 285.3, 533.7, and 102.7 eV in C(1s), O(1s), and Si(2p) spectra, respectively. The additional peaks indicate the covalent bonding between the silanol group and Si wafer, which suggests that the homogeneous distribution of SBR-functionalized silica nanoparticles within the matrix comes from the chemical interaction between silica nanoparticles and end-functionalized SBS polymers.

**Figure 11:**
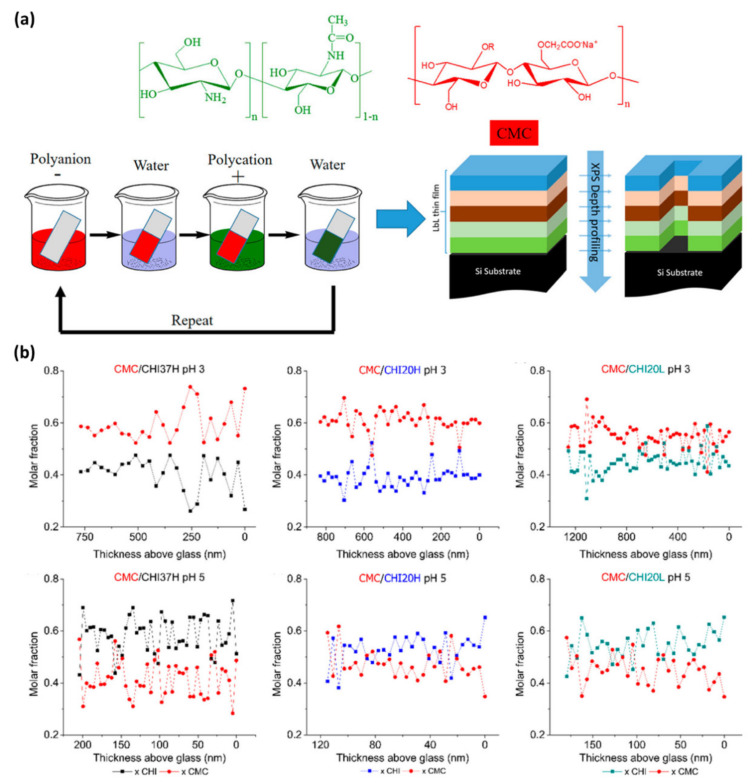
(**a**) Schematic illustration of preparation of layer-by-layer method using chitosan and XPS depth profiling. (**b**) Depth profiling of CMC/CHI systems (Reproduced with permission from Reference [59]; Copyright (2018) American Chemical Society).

Rubner and Cohen et al. utilized depth profiling XPS analysis for the investigation of the internal chemical composition within chitosan-based nanocomposite films [59]. The composite films were prepared by the layer-by-layer method using chitosan (CHI) and carboxymethylcelluose (CMC) as the polycation and polyanion, respectively (Figure 11a). CHI possessed a different degree of acetylation and molecular weight produced by the ultrasound-assisted deacetylation reaction. Repetition of C_60_^+^ cluster-ion sputtering and XPS analysis provided the internal composition of the composite films. By the depth profiling technique, the molar fraction of CHI/CMC was obtained by the mass balance of nitrogen and carbon. As a weak polyanion, the charge density of the CMC dependent on the medium pH: de-protonated and high negative charge at pH 5 and protonated and low negative charge at pH 3. CHIs have similar surfaces charge at the range between pH 3 and 5, due to the amino groups of CHI having pKa ~6.5. At the constant degree of acetylation, the electrostatic interaction between CHI and CMC resulted in a well-controlled growth rate per cycle and rich CHI composition within the composite films at pH 5. The less amount of anionic charge of CMC at pH 3 resulted in poor CHI and rich CMC composition to balance the counter-charges. The degree of acetylation of CHI affected the internal components in the same way. The higher the degree of acetylation, the lower the positive charge of the CHI. At the same pH, CHI with a higher degree of acetylation led to rich CHI composition to balance the negative charge of CMC. This study was an example for quantitative and depth profiling analysis of XPS to characterize the effect of fabrication parameters on the composition of composite materials.

## 3. Energy-Dispersive X-ray Spectroscopy

**Figure 12:**
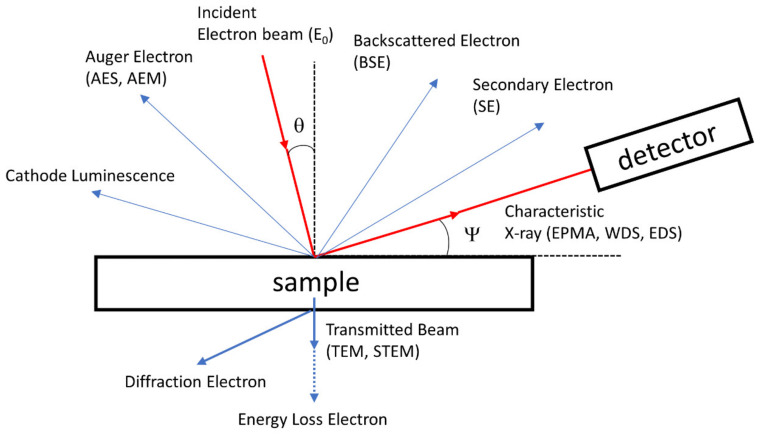
Possible routes for electron beam to interact with a sample and their consequences.

Energy-dispersive X-ray spectroscopy (EDS, EDX, or EDXS) is a widely utilized non-destructive spectroscopic technique for both qualitative and quantitative analyses to find chemical substances and compositions of a variety of materials using the interaction of provided electron beam with a material [60,61,62,63,64,65,66,67,68,69]. After the excitation of electrons in the material with an electron beam, an X-ray with specific energy is emitted when the excited electrons drop to the ground state (Figure 12). Since the energy of the emitted X-ray of each element is different, the type of elements in the material can be identified by measuring the energy of emitted X-rays. Typically, elements in a range of the atomic number of 11 (sodium) to 92 (uranium) can be qualitatively and quantitatively analyzed in the concentration of from near 1% to 100% [70]. Furthermore, it is well-established as an easily accessible tool attached to electron microscopies such as scanning electron microscopy (SEM) and transmission electron microscopy (TEM): during imaging with the microscopies, the EDS analysis can be simply and quickly conducted with spatial element mapping in conjunction with a microscopic image, without any need for specific sample pre-treatments for EDS [71,72,73]. Very thin conductive metal or carbon layer coating of a nonconductive sample is often necessary for electron microscopic imaging; although the sampling depth of EDS is about few microns, the acquired EDS spectrum should be carefully interpreted. Notably, the distribution of some components in the whole material often affects the final various properties of the polymer composites, meaning that EDS becomes a more important tool for polymer nanocomposite science and engineering. In this section, we will briefly discuss how this analysis technique has been involved in the characterization of polymer nanocomposite.

**Figure 13:**
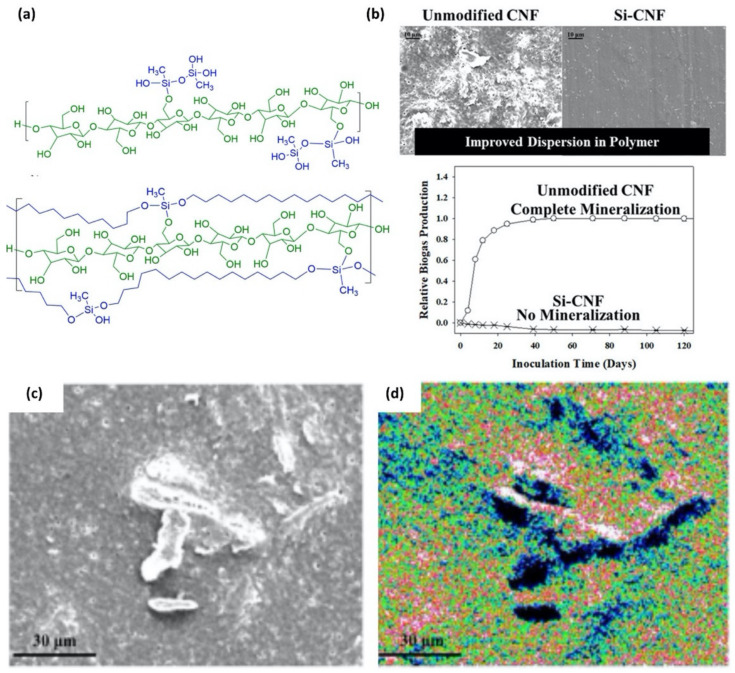
(**a**) Chemical structures of cellulose nanofibrils (CNFs) with the siloxane coating (blue) on the CNF (green) through covalent bond. (**b**) SEM image of the nanocomposite surface (top) and biogas production capability (bottom) for unmodified CNFs and siloxane coated CNFs. (**c**) SEM image and (**d**) energy-dispersive X-ray spectroscopy (EDS) oxygen mapping of an aggregation of unmodified CNFs in polyhydroxyalkanoates (white color representing high oxygen concentration). (Reproduced with permission from Reference [97]; Copyright (2018) American Chemical Society).

The dispersibility of a specific nanomaterial in a polymer matrix is one of the key issues in nanocomposite sciences. To improve the homogeneity of a filler nanomaterial in the matrix, several strategies such as chemical modification of the filler material [74,75], core-shell strategy [76,77,78], ultra-sonication [79], aerosol [80,81,82,83,84,85], and crosslinking [86,87,88,89,90] have been proposed. In this aspect, the chemical modification of organic filler materials has been extensively investigated [91,92,93,94,95,96]. For example, cellulose, abundant, affordable, and sustainable natural polymer, has attracted attention due to a large amount of the hydroxyl group, which not only forms many interchain hydrogen bonds, yielding great physical properties, but can also be exploited for chemical modification to improve the interaction with the polymer matrix. Frank et al. recently showed a surface modification of cellulose nanofibrils (CNF) with methyl trimethoxysilane, propyl trimethoxysilane, and aminopropyl trimethoxysilane [97] (Figure 13). Condensation polymerization of the silane compound on the CNF surface led to the formation of a thin siloxane layer without altering the structure and crystallinity of CNF. The silanization increased the dispersibility and stability of CNF in organic solvents and suppressed the mineralization of CNF, which largely changes the properties of fibrils as a filler [98]. The quantitative analyses with EDS provided the amount of silane attached to the CNF surface: for example, the silanization with 0.1 wt% of methyl trimethoxysilane resulted in the incorporation of approximately one silicon-containing unit on one repeating unit of the cellulose. Furthermore, in a nanocomposite comprising the CNF and polyhydroxyalkanoates, the combination of SEM and elemental mapping with EDS highlights the importance of the surface coating for improvement of the dispersibility and stability of CNF in the polymer matrix.

**Figure 14:**
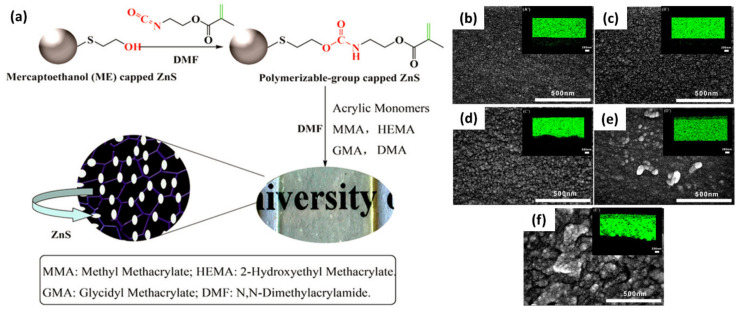
(**a**) Nanocomposite fabrication using ZnS nanoparticles surface-anchored with polymerizable group. (**b**–**f**) SEM images of hydrogel with different concentration of the ZnS nanoparticles, and EDS sulfur mapping (insets) for each nanocomposite hydrogel: (**b**) 50 wt%, (**c**) 60 wt%, (**d**) 70 wt%, and (**e**) 80 wt% of polymerizable-group-capped ZnS nanoparticles, and (**f**) 50 wt% physically embedded ME-capped ZnS. (Reproduced with permission from Reference [107]; Copyright (2018) American Chemical Society).

For functional inorganic material/polymer nanocomposites, an elemental mapping with EDS is a powerful means to evaluate the homogeneity of the resulting composite materials [99,100,101,102,103,104,105,106]. Figure 14 shows the fabrication of high refractive index nanocomposite hydrogel by copolymerizing N,N-dimethyl acrylamide (DMA), 2-hydroxyethyl methacrylate (HEMA) or glycidyl methacrylate (GMA) or methyl methacrylate (MMA), and polymerizable methacrylate grafted on ZnS nanoparticle [107]. The properties of a hydrogel such as permeability, mechanical robustness, water content, and refractive index can be effectively controlled by simply varying the amount of ZnS capped with a polymerizable functional group. The impact of the presence of the chemical attachment of ZnS on the network was clearly revealed using elemental mapping with EDS: a heterogeneous distribution of sulfur was observed in the nanocomposite formed with physically embedded ZnS nanoparticles without polymerizable surface functionality, while sulfur was homogeneously distributed in the hydrogel with chemically attached ZnS particles.

**Figure 15:**
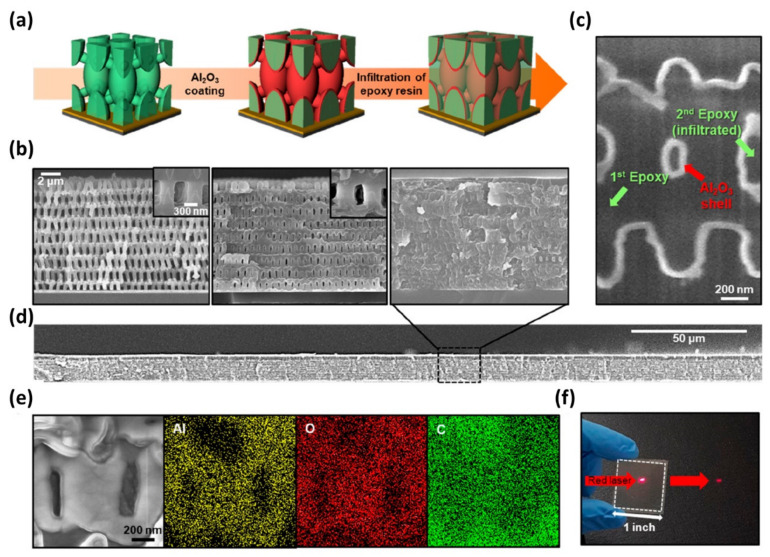
(**a**) Schematic illustration of the fabrication of 3D nanocomposite structure fabrication. (**b**) SEM cross-sectional image at each step. (**c**,**d**) Cross-section SEM image by intensive ion beam (FIB) of (**c**) high- and (**d**) low-magnification cross-section. (**e**) EDS mapping images of Al, O, C elements for the 3D nanocomposite. (**f**) Photographic image confirming transparency of the fabricated structure. (Reproduced with permission from Reference [24]; Copyright (2018) American Chemical Society).

The simultaneous use of EDS with SEM enables a close examination of the chemical composition in a three-dimensional (3D) structure, which is non-trivial with other characterization techniques. Ahn et al. reported the fabrication of unconventional inorganic/organic polymer nanocomposite with continuous ceramic nanofiller [24] (Figure 15). The nanocomposite material containing ceramic nanofillers exhibits outstanding physical properties such as high toughness, elastic moduli, and yield strength. For homogeneous distribution of components, the authors demonstrated the processes consisting of proximity field nanopatterning of epoxy resin, atomic layer deposition of Al_2_O_3_ on the surface of a pre-defined epoxy structure, and filling of epoxy resin. This approach realized the well-defined location of the inorganic filler material in a polymeric matrix to avoid aggregation of fillers that degrades optical properties. However, the confirmation of the epoxy/Al_2_O_3_/epoxy structure is challenging; the use of EDS and SEM effectively showed that the epoxy is separated uniformly based on Al_2_O_3_ nanolayer, highlighting its usefulness for structural characterization of small 3D objects and ultimately, comprehensive understanding of structure-property relationships.

## 4. X-ray Absorption Spectroscopy

Near-edge X-ray absorption fine structure (NEXAFS), also known as X-ray absorption near edge structure (XANES), is a spectrometric method measuring the spectrum of the edge portion where X-ray is absorbed in a target sample [108]. When an X-ray is illuminated, electrons residing in orbitals near the atomic nucleus are typically excited due to the high energy of the X-ray. The irradiated X-ray excites core electrons in a sample to continuum empty states right above the vacuum level, or to unoccupied electron orbitals, i.e., Rydberg states, below the vacuum level. Generated empty 1s orbitals upon the process are filled with other electrons in orbitals at higher energy levels and simultaneously, a certain amount of energy corresponding to the transition is released. This energy is transferred to other electrons, allowing their emission to the external environment. NEXAFS signal is generated by the resonance or interference between electron waves emitted by that process and backscattered electron waves by other electrons of surrounding atoms. The signal of the emitted photoelectrons with specific energy between 1s orbital level and the excited level is shown as an intense peak in the NEXAFS spectrum [109]. Due to the nature of absorption spectroscopy, NEXAFS requires the variation of X-ray energy and therefore, a single X-ray source cannot be utilized. Typically, a synchrotron X-ray source is necessary for full spectrum measurements with high resolutions. 

In cases of organic/polymeric materials, electrons in C(1s) orbital are easily excited to σ* orbital, π* orbital, and Rydberg state, K-edge spectrum of carbon is typically examined to identify elements and chemical bonding in a material. The greatest advantage of NEXAFS is that both qualitative and quantitative analyses are feasible for characterizing the orientation and composition in a sample material in a range of approximately 10 nm of sampling depth [110,111,112,113,114,115,116,117,118,119]. Notably, for interpreting NEXAFS spectra obtained from organic materials, the building-block model is often employed. In the model, the spectrum is the result of the summation of the contribution of local subunits in the system [120], which is relevant to the system comprising large molecules comprising certain repeating units, i.e., polymers. In this section, the use of NEXAFS for the characterization of polymer nanocomposite for the studies of the physical state of functionalities in polymeric components at the interface as well as the surface chemical composition of the materials.

### 4.1. Identification of Chemical Environments and Compositions

Similar to XPS, the NEXAFS spectrum is sensitive to the types of chemical bonding, and specific chemical functionalities can be easily identified by observing the peak of the target element. Urquhart et al. showed a systematic variation of the peak in C(1s) and O(1s) NEXAFS spectra for different polymeric materials, e.g., polycarbonate, polyurethane, polyurea, poly(ethylene succinate), poly(methyl methacrylate), polyamide, and poly(vinyl methyl ketone) (tabulated in Table 2) [121]. Considering the characteristic chemical bonding in the materials, they found that peaks exhibited by the transitions of C(1s) and O(1s) electrons to π*_C=O_ are highly sensitive to the chemical environment; the peak position tends to shift to higher energy when the examined element is bound to other elements with higher electronegativity. Table 3 tabulates the peak position observed from various chemical functionalities [118]. The composition of a composite consisting of two or more polymer components can be quantitatively studied using NEXAFS, for example, a surface composition of a composite material consisting of two functional conjugated copolymers, poly(9,9′-dioctylfluorene-*co*-*bis*-N,N’-(4-butyl phenyl)-*bis*-N,N’-phenyl-1,4-phenylene-diamine) (PFB) and poly(9,9′-dioctylfluorene-*co*-benzothiadiazole) (F8BT) [115]. The difference in the chemical structure allows us to observe two distinct transitions of C(1s) electrons to π*_C=C_, and the intensities of the peaks were utilized to determine the surface composition. This is relevant to understand the effect of the surface composition on the performance of the composite towards a target application, in this case, the improvement in efficiency of an organic photovoltaic device.

**Figure 16:**
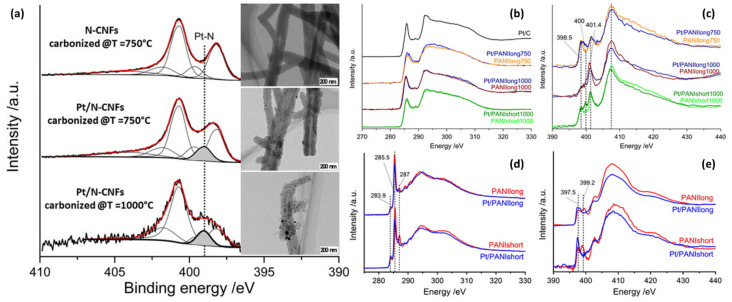
(**a**) XPS spectra and TEM images, and near-edge X-ray absorption fine structure (NEXAFS) spectra of (**b** and **d**) C(1s) K-edge, (**c** and **e**) N(1s) K-edge nanofiber samples made from PANI. (Reproduced with permission from Reference [126]; Copyright (2016) American Chemical Society).

The capability of NEXAFS to examine chemical states in composite materials offers an outstanding route to probe doping in the materials, which is relevant for electronic and electrochemical applications [122,123,124,125]. Figure 16 displays a nanocomposite of Pt in PANI nanofiber, in which a metal catalyst such as Pt nanoparticle in carbon nanofiber prepared from a conjugated polymer such as polyaniline (PANI) was used for an electrochemical oxygen reduction reaction [126]. For the electrochemical reaction, the interaction between Pt and the polymer, the-so-called doping, is important to actively transfer electrons in the system so that the electrochemical reaction is facilitated. The interaction can be examined by observing the NEXAFS spectra of the system as the peak of nitrogen is shifted due to the changes in its electronic density (Figure 16), when it is adjacent to the surface of Pt nanoparticles. This highlights the significance of NEXAFS for probing electronic structure and behaviors which highly affect its final performance in desired applications.

### 4.2. Physical States of Interfaces of Two Components in Nanocomposites

**Figure 17:**
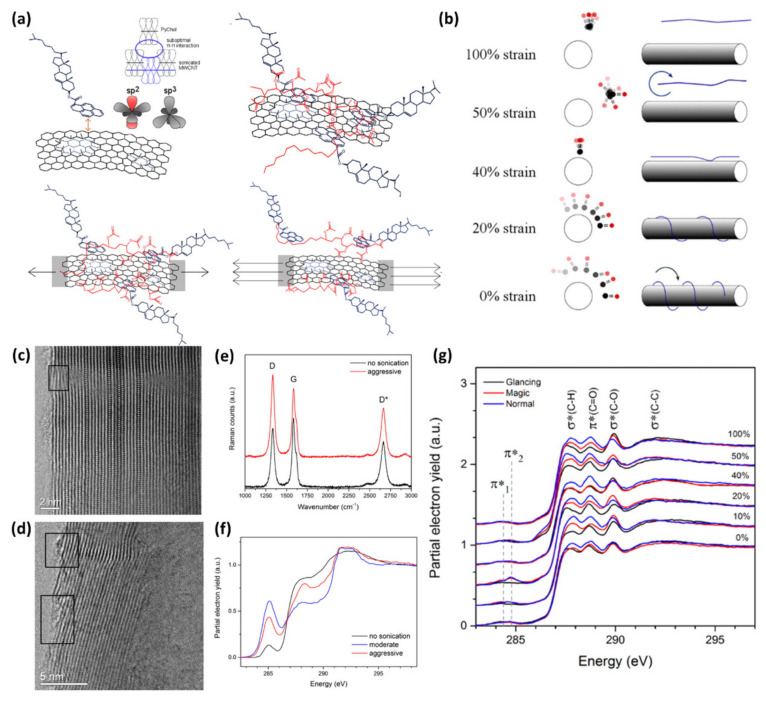
(**a**) Schemes showing the interaction of PyChol to carbon nanotube in ethylene-vinyl acetate (EVA) matrix at low or high strain. (**b**) “Stick and slip” mechanism with the increase in strain. (**c**) TEM images of non-sonicated and (**d**) sonicated multiwall carbon nanotubes (MWCNTs). (**e**) Raman spectra of non-sonicated and aggressively sonicated MWCNTs. (**f**) NEXAFS spectra of non-sonicated, moderately sonicated, and aggressively sonicated MWCNTs. (**g**) Angle-resolved C(1s) K-edge NEXAFS spectra with the increase in strain applied to the nanocomposite. (Reproduced with permission from Reference [133]; Copyright (2015) American Chemical Society).

The extraordinary sensitivity of NEXAFS to chemical bonding and element enables us to specifically observe the most characteristic part in the system for revealing the physical states, i.e., the order and orientation on surfaces and interfaces, of the molecules and functional groups in a particular interest [127,128,129,130,131]. Observing polarization dependence of a characteristic peak of a particular functional group in a composite material enables close examination of the orientation. As a typical example, NEXAFS studies of composite materials comprising pyrene-tethered azobenzene molecules on graphene were conducted to investigate the detailed molecular orientations of the molecules bound to graphene via π−π interactions [132]. The azo π* peak at 399 eV showed a clear angular dependence which was translated into a molecular tilt angle of ~30°. These results are of importance to gain a deep understanding of the previous observation of effective p-doping of graphene by the azobenzene molecules. Besides graphene or graphite, a variety of carbon materials such as carbon nanotubes are also widely utilized in nanocomposite materials. One of the issues in the carbon materials for nanocomposites is to achieve a homogeneous distribution of carbon fillers in a polymer matrix. To improve the dispersibility, it is important to comprehend how the dispersion agent (e.g., surfactants and compatibilizers) and polymer matrix interacts with the surface of carbon materials, and the comprehension can be attained using NEXAFS. Winter et al. implemented Raman and NEXAFS spectroscopies for the effects of aggressive sonication while monitoring the molecular arrangement of surfactants in the dispersion of multiwall carbon nanotubes (MWCNT) to prepare MWCNT/ethylene-vinyl acetate (EVA) polymer nanocomposites (Figure 17) [133]. The results suggest the damages in MWCNT by aggressive sonication. More importantly, in EVA/MWCNT composite systems, the angular dependence of π*_C=O_ peak at 288.6 eV shown in Figure 17g reveals the conformation changes in polymer chains that surround MWCNT: when the stain is applied to the system, the unlatching of the polymer from MWCNT occurs by “stick and slip” and subsequent “backbone twisting” mechanism. Since the polymer conformation in the composite can be mechanically modulated, precise control and processing of specific composite systems such as photovoltaic systems become possible, in which the conformation of conjugated backbone largely affects power conversion efficiency.

**Figure 18:**
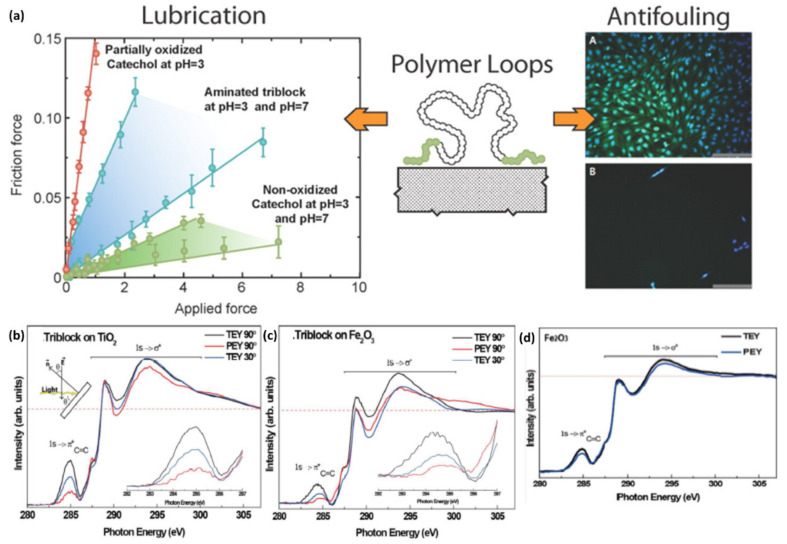
(**a**) Friction force of the triblock copolymer chain with different functional groups (left), the schematic showing triblock copolymer adsorbed onto the surface of mica (middle), and optical microscopic images (right) showing the adhesion of cells to the bare glass (top) and glass coated with a catechol-functional-triblock copolymer (bottom). NEXAFS spectra of (**b**) TiO_2_ and (**c**) Fe_2_O_3_ substrates adsorbing the triblock copolymer (partial electron yield (PEY) mode at incidence angles of 90° and 30°). (**d**) NEXAFS spectra in total electron yield (TEY) and PEY mode of the triblock copolymer coated on Fe_2_O_3_ surface (Reproduced with permission from Reference [136]; Copyright (2016) American Chemical Society).

For surface-active composite materials, NEXAFS provides the physical conformation of the surface functionalities so that the mechanism of surface events is induced by the functionalities [113,134,135]. Anti-fouling and fouling-release surfaces, one of the common, but relevant applications, are an excellent example. Kang et al. demonstrated the interface of the polymer and the solid surface where a triblock copolymer consisting of two end blocks with a catechol anchoring group and the looping poly(ethylene oxide) (PEO) midblock is adsorbed [136]. Due to strong adhesion capability of catechol group on the surface, PEO loops are strongly fixed to show effective high lubrication and low specific adhesion of organisms relative to the random coils or brush polymers, suggestive of the feasibility to develop high-performance biomedical coatings. The key factor, the loop conformation of the triblock copolymer on the surface of mica, was examined using NEXAFS studies. They used two data collection methods, total electron yield (TEY; sampling depth of more than 10 nm) and partial electron yield (PEY, sampling depth of approximately 1 nm) modes. Comparison of PEY and TEY spectra, they found that the C (1s) π* peak in PEY mode showed lower intensity than C (1s) σ* peak at 288.5 eV (C-O) in PEY mode, and far lower intensity than the π* peak from the TEY mode at the same angle. (Figure 18) These results suggest the inhomogeneous distribution of the catechol group in the z-axis on the surface with a higher concentration near the surface. Therefore, the middle PEO block is not latched on the surface, but rather forms the loop structure. These studies highlighted the capability of NEXAFS to probe the interface where the polymer interacts with solid surfaces, which has been one of the significant research areas in composite science and engineering.

## 5. New Opportunity with Emerging X-ray Based Techniques Enabling Near Ambient Pressure Measurements

Conventionally, XPS, EDS, and NEXAFS techniques are performed under ultra-high vacuum (UHV) conditions as electrons are easily scattered by inelastic interactions with gas molecules. As a consequence, acquired chemical and physical information cannot be related to the characteristics and chemical changes of the materials under realistic conditions. In recent decades, technical and instrumental advances to overcome this limitation have been demonstrated by the improvement in electron focusing and detecting ability [137], enabling access to the pressure limit to the range of tens-hundreds mbar level: namely near ambient pressure (NAP) XPS and NEXAFS [138], and variable pressure (VP) SEM/EDS [139]. With these advances, a close examination of various surfaces interacting with a variety of gas molecules including water vapor and reactive gases became possible with real time measurements. The applicability is not limited to fundamental molecule–surface interaction studies: catalytic system [137,140,141], electrochemical reaction occurring on an electrode surface [142], surface reaction kinetics [137,143,144], the effect of the presence of relevant gas molecules such as water to the performance of a specific material system [138,145]. Initially, they were available with only synchrotron X-ray sources; however, it was recently shown that a laboratory system with typical X-ray sources is capable of decent acquisition of NAP-XPS spectra, suggesting its potential to be utilized for the quality control of the production process in industries [146,147]. 

Notably, these techniques are predominantly implemented for inorganic materials systems due to their applicability to inorganic surfaces, which is relevant for catalyst and energy applications. For the system comprising organic components, NAP-XPS has been utilized mainly among the techniques. One of its advantages is the suppression of the charging effect that is often observed in the measurements of nonconductive organic surfaces, e.g., poly(tetrafluoroethylene) [146]. The interaction between gas molecules and polymeric materials is also possible: for example, the interaction of water to hydrophilic polymer chains in superabsorbent hydrogel such as a soft contact lens can be probed effectively [146]. In the nanocomposite systems, a lower number of studies using NAP-XPS can be found in the literature regarding the interaction of relevant molecules with inorganic fillers on or near the surface of the nanocomposite, e.g., the chemical state of Pt in Pt/C electrodes under humid condition [148]. Probing the interaction at organic/inorganic interfaces was recently attempted: Pletincx et al. investigated the mechanism of the interactions among three components of water vapor, poly(methyl methacrylate) (PMMA) chain, and aluminum oxide surface [149]. Careful in situ examination of C(1s) and O(1s) NAP-XPS spectra suggested that water played an important role to induce hydrolysis of methyl ester group in PMMA to carboxylic acid, which is relevant for the strong adsorption of the polymer chain on the oxide surface [150]. 

NAP X-ray techniques provide a kinetic insight at a molecular level on a specific surface. In polymer nanocomposite science and engineering, these frontier techniques will apparently offer a significant handle as the chemical and physical changes of the polymer nanocomposite materials under realistic environments should be deeply comprehended for improving the performance of the composites in target applications. 

## 6. Concluding Remarks

We have discussed the summary of three important spectroscopic techniques implementing the photoelectric effect, absorption, and emission of or by X-ray: XPS, EDS, and NEXAFS. While these spectroscopic methods have been extensively employed to analyze a variety of composite material structures, fundamental principles and additional techniques should be thoroughly apprehended towards deeper understandings at a molecular level. On this firm foundation, these techniques allow us to access relevant information: qualitative and quantitative chemical compositions such as the relative and absolute concentrations of elements of interests and even the relative concentration of specific chemical bonds near the surface of or deep inside a given material. More importantly, these techniques enable us to closely examine the interface of a matrix polymer and a filler material in the nanocomposite, which is the key part of understanding why specific material properties are improved. From this perspective, X-ray based techniques allow us to acquire the chemical composition near the surface and the information on the molecular orientation of small molecules or macromolecules on the surface. In this process, the studies on organic thin films on different solid substrates should not be underestimated as they can provide simplified systems towards a better understanding of complex nanocomposite systems. Ultimately, the advances in these techniques will lead us to learn the complete structure-properties relationships required for rational design, realization, full characterization, and application of advanced nanocomposite materials which can open up novel applications.

## Figures and Tables

**Table 1:** Representative XPS C(1s) binding energy of different functional groups.

Functionality	Binding Energy (eV)
C-H, C-C (hydrocarbon)	285.0
C-N (amine)	286.0
C-O-H, C-O-C (alcohol, ether)	286.5
C=O (carbonyl)	288.0
N-C=O (amide)	288.2
O-C=O (acid, ester)	289.0
N-(C=O)-N (urea)	289.0
O-(C=O)-N (carbamate)	289.6
O-(C=O)-O (carbonate)	290.3
C-Cl (Cl with carbon)	286.5
C-F (F with carbon)	287.8
-CH_2_CF_2_- (2F with carbon)	290.6
-CF_2_CF_2_- (PTFE)	292.0
-CF_3_- (3F with carbon)	293–294

**Table 2:** NEXAFS energy of 1s→π*_C=O_ transition in carbonyl cores observed from different polymers.

Polymer	C(1s) Energy (eV)	O(1s) Energy (eV)
polycarbonate	290.4	532.9
polyurethane	289.9	N/A
polyurea	289.5	N/A
poly(ethylene succinate)	288.6	532.2
poly(methyl methacrylate)	288.5	532.1
nylon-6	288.2	532.2
poly(ethylene terephthalate)	288.15	531.5
poly(vinyl methyl ketone)	286.6	531.3

**Table 3:** NEXAFS transition energy of electrons of C(1s) to π* molecular orbital and typically observed peak position of common chemical functionality (Reproduced from Reference [118] with permission from Elsevier).

eV	Transition	Functionality
283.7	1s→π*	Quinone
	1s→π*	Protonated/alkylated
284.9–285.5	1s→π*	Aromatic and PNA Carbonyl substituted
285.8–286.4	1s→π*	Aromatic, Phenolic C-OH Ketone
287.1–287.4	1s→π*	Aliphatic carbonyl
287.7–288.3	1s→π*	Aromatic carbonyl C=O
287.6–288.2	1s→3p/σ*	CH_3_, CH_2_, CH
288.2–288.6	1s→π*	COOH
289.3–289.5	1s→3p/σ*	C-OH, alcohol
